# Assessment of Atrial Conduction Times in Patients with Newly Diagnosed Parkinson's Disease

**DOI:** 10.1155/2018/2916905

**Published:** 2018-07-16

**Authors:** Yiğit Çanga, Ayşe Emre, Gülbün Asuman Yüksel, Mehmet Baran Karataş, Nizamettin Selçuk Yelgeç, Ufuk Gürkan, Ali Nazmi Çalık, Hülya Tireli, Sait Terzi

**Affiliations:** ^1^Department of Cardiology, Dr. Siyami Ersek Cardiovascular and Thoracic Surgery Center, Istanbul, Turkey; ^2^Department of Neurology, Haydarpasa Numune Training and Research Hospital, Istanbul, Turkey

## Abstract

**Background:**

An increased risk of ischemic stroke has been reported in patients with Parkinson's disease (PD). Atrial fibrillation (AF) is strongly associated with ischemic stroke. Prolonged atrial electromechanical delay (EMD) is an independent predictor for the development of AF.

**Aims:**

The aim of the present study was to evaluate the atrial conduction parameters in patients with PD and to assess their relation with the severity of PD.

**Study design:**

We prospectively enrolled 51 consecutive patients with newly diagnosed PD and 31 age- and sex-matched non-PD subjects.

**Methods:**

To assess atrial electromechanical coupling (PA), the time intervals from the onset of p wave on ECG to the late diastolic wave at the septal (PAs) and lateral (PAl) mitral annulus and lateral tricuspid annulus (PAt) were measured on Tissue Doppler Echocardiography (TDE). The difference between PAs-PAl, PAs-PAt, and PAl-PAt were defined as left intra-atrial, right intra-atrial, and interatrial EMD, respectively. P-wave dispersion (PWD) was calculated from the 12-lead ECG.

**Results:**

PWD, PAs, PAl, and PAt durations were significantly prolonged in the PD group (all *p* < 0.001). Interatrial, right, and left intra-atrial EMD were also significantly longer in PD patients (*p* < 0.001, *p* < 0.001 and *p*=0.002, resp.). There were significant positive correlations between disease severity (UPDRS score) and PWD (*r*=0.34, *p*=0.041), left intra-atrial (*r*=0.39, *p*=0.005), and interatrial EMD (*r*=0.35, *p*=0.012). By multivariate analysis, PWD (OR: 1.13, 95% CI: 1.02–1.25; *p*=0.017), LA volume index (OR: 1.19, 95% CI: 1.02–1.37; *p*=0.021), left intra-atrial (OR: 1.12, 95% CI: 1.01–1.24; *p*=0.041), and interatrial EMD (OR: 1.08, 95% CI: 1.01–1.16; *p*=0.026) were found as independent predictors of PD.

**Conclusion:**

Atrial conduction times were longer and correlated with the severity of disease in PD patients. Prolonged inter- and intra-atrial-EMD intervals were also found as independent correlates of PD. These findings may suggest an increased predisposition to atrial fibrillation in PD.

## 1. Introduction

Parkinson's disease (PD) has been associated with an increased risk of ischemic stroke and stroke-related mortality [1–3]. A recent population-based, propensity score-matched longitudinal follow-up study demonstrated that newly diagnosed PD was related with an increased risk of developing ischemic stroke [4]. Patients with atrial fibrillation have about 3- to 5-fold higher risk of stroke even after adjustment for risk factors [5]. Atrial fibrillation has been associated with stroke in different patient populations [6]. Prediction of atrial fibrillation may be crucial for risk stratification of PD patients with regard to ischemic stroke.

Prolonged atrial conduction times have been related to both onset and recurrence of atrial fibrillation [7, 8]. Tissue Doppler Echocardiography (TDE) has been used to determine atrial electromechanical coupling and electromechanical delay (EMD) intervals between different regions as indicators of electrical and/or structural remodeling of atria and as predictors of atrial fibrillation [9]. Regional changes in atrial conduction times might have a different influence on surface p waves leading to an interlead variation in p-wave duration called p-wave dispersion (PWD) [10]. In the present study, we investigated atrial conduction parameters in patients with newly diagnosed PD and also evaluated their relationship with the severity of PD.

## 2. Methods

### 2.1. Study Population

Fifty-one consecutive patients with newly diagnosed PD and 31 age- and sex-matched non-PD subjects were prospectively enrolled between January 1st, 2015 and December 31, 2015. Patients with PD were diagnosed according to the UK Parkinson's Disease Society Brain Bank Criteria [11]. The severity of PD was assessed by Unified Parkinson's Disease Rating Scale (UPDRS) [12]. Patients with a previous diagnosis of PD, stroke, other extrapyramidal disease, abnormal movement disorder, cerebral degeneration, cardiac arrhythmia, valvular heart disease, heart failure, coronary artery disease, chronic obstructive pulmonary disease, chronic renal failure, thyroid disease, active infectious disease, poor echocardiographic image quality, and a history of cardiac surgery or implanted device were excluded from the study. Baseline history, medication, and electrocardiography were recorded at the beginning of enrollment. PWD was defined as the difference between the maximum and minimum p-wave duration from multiple surface ECG leads [10]. The study was approved by the local ethics committee. Written informed consents were obtained from all participants.

### 2.2. Echocardiography

All patients were evaluated by transthoracic M mode, two dimensional, pulsed wave, continuous wave, colour flow, and TDE using the GE Vivid 3 system (GE Vingmed, Horten, Norway) with a 2.5–3.5 MHz transducer. Continuous single lead ECG was obtained from each participant during echocardiography.

LV diameter and wall thickness were measured by M-mode echocardiography. LV ejection fraction was calculated by Simpson's method according to the American Society of Echocardiography guidelines [13]. The mitral valve inflow pattern (E wave, A wave, E-wave deceleration time, E/A ratio, and isovolumic relaxation time) was measured by pulsed wave Doppler. LA volume was obtained from apical four and two chamber views by a disc method and indexed to body surface area [13].

TDE was performed by adjusting the pulsed Doppler signal filters to acquire the Nyquist limit of 15–20 cm/s and using the minimal optimal gain. Motions were recorded simultaneously with electrocardiogram in lead II to assess the relation between atrial electrical phases and myocardial motion. To assess atrial electromechanical coupling (PA), the time intervals from the onset of p wave on ECG to the late diastolic wave at the septal (PAs), and lateral (PAl) mitral annulus and lateral tricuspid annulus (PAt) were measured on TDE. The difference between PAs-PAl, PAs-PAt, and PAl-PAt were defined as left intra-atrial, right intra-atrial and interatrial EMD, respectively [7, 9]. Echocardiographic measurements were performed by two cardiologists. Patients with *a* > 5% difference between the measurements of two cardiologists were not included.

### 2.3. Statistical Analysis

Statistical analysis was performed using SPSS 16.0 (SPSS, Inc. Chicago, Illinois). A two-tailed *p* value < 0.05 was considered statistically significant. Categorical variables were expressed as frequencies (percentages). Continuous variables were presented as mean ± standard deviation (tested for normality with Shapiro–Wilk test). Categorical variables were compared using the chi-square or Fischer's exact tests. Group means for continuous variables were compared using independent sample *t*-test. Pearson's correlation test was performed to assess the correlation between atrial conduction times and the severity of PD. Multivariate regression analysis was used to identify independent predictors of PD. Age, gender, hypertension, diabetes, LA volume index, PWD, and EMD intervals were included in the multivariate models.

## 3. Results

Fifty-one consecutive PD patients (mean age: 66.3 ± 12.4 years and 71% men) and 31 non-PD subjects (mean age: 69.8 ± 12.7 years and 52% men) entered the study. Mean UPDRS score of the PD group was 35.3 ± 17.9 (range, 10 to 75). Baseline demographic and clinical characteristics of the PD and non-PD groups are provided in Table 1. There was no statistically significant difference in all the baseline characteristics between the PD and non-PD groups.

Echocardiographic parameters are provided in Table 2. LA volume index was significantly higher in the PD group (*p*=0.006). Mitral E/A was lower and mitral E-wave deceleration time was higher in the PD group, but both failed to reach statistical significance (*p*=0.057 and *p*=0.058, resp.). The remaining standard echocardiographic parameters were comparable between the two groups.

Atrial conduction time intervals are shown in Table 2. PWD, PAs, PAl, and PAt durations were significantly prolonged in the PD group (all *p* < 0.001). Left intra-atrial, right intra-atrial, and interatrial EMD were significantly longer in PD patients (*p* < 0.001, *p* < 0.001, and *p*=0.002, resp.). PWD showed significant correlations with left intra-atrial (*r*=0.57, *p* < 0.001) and interatrial EMD (*r*=0.54, *p* < 0.001).

**Figure 1:**
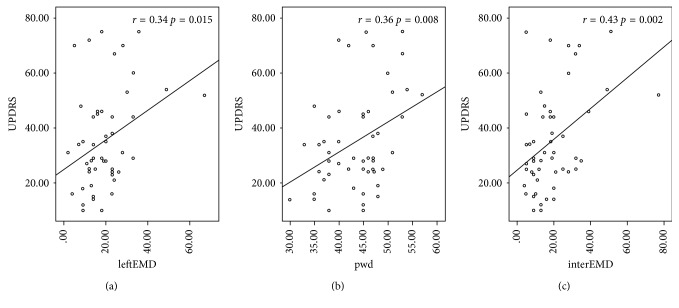
(a) Correlation between left intra-atrial EMD and the severity of Parkinson's disease (UPDRS score). (b) Correlation between p-wave dispersion and the severity of Parkinson's disease. (c) Correlation between interatrial EMD and the severity of Parkinson's disease UPDRS, Unified Parkinson's Disease Rating Scale; EMD, electromechanical delay.

There were significant positive correlations between disease severity (UPDRS score) and PWD (*r*=0.36, *p*=0.008), left intra-atrial (*r*=0.34, *p*=0.015), and interatrial EMD (*r*=0.43, *p*=0.002) (Figure 1). By multivariate analysis, PWD (OR: 1.13, 95% CI: 1.02–1.25; *p*=0.017), LA volume index (OR: 1.19, 95% CI: 1.02–1.37; *p*=0.021), left intra-atrial (OR: 1.12, 95% CI: 1.01–1.24; *p*=0.041), and interatrial EMD (OR: 1.08, 95% CI: 1.01–1.16; *p*=0.026) were found to be independent predictors of PD (Table 3).

## 4. Discussion

The major findings of the present study are (1) patients with newly diagnosed PD are more likely to have abnormal atrial conduction times as assessed by prolonged PWD, PAs, PAl, PAt, intra-atrial, and interatrial EMD; (2) prolonged atrial conduction times were significantly correlated with the severity of PD as assessed by the UPDRS score, and (3) PWD, left intra-atrial, and interatrial EMD were found to be independent predictors of PD.

Atrial fibrillation is the most common sustained arrhythmia in clinical practice [14]. Electrophysiological studies have revealed prolonged atrial conduction times as predictors of atrial fibrillation [15, 16]. In a previous study examining atrial conduction times with TDE, Deniz et al. have found significant correlations regarding left intra-atrial and interatrial conduction times detected by TDE and by electrophysiological study [9]. PWD and left intra-atrial conduction time detected by TDE were found to be independent predictors of inducibility of sustained atrial fibrillation in their study. Sequential analysis of atrial electromechanical coupling to evaluate the mechanisms of paroxysmal atrial fibrillation showed that atrial electromechanical coupling at the interventricular septum, left lateral mitral annulus, and right lateral tricuspid annulus was significantly longer in patients with paroxysmal atrial fibrillation (with or without underlying heart disease) compared to control subjects [7]. Left intra-atrial EMD was significantly prolonged even after correction for age in patients with atrial fibrillation [7]. The juxtaposition of atrial fibrotic lesions with normal atrial fibers has been suggested as a mechanism for nonhomogeneity of atrial conduction in atrial fibrillation [17]. Prolongation of atrial electromechanical coupling might be due to the time delay from atrial electric activation to myocardial contraction and/or left atrial enlargement [8]. In our study, PAs, PAl, and PAt durations were significantly prolonged and interatrial, right intra-atrial, and left intra-atrial EMD were significantly longer in patients with newly diagnosed PD. LA volume index was also increased in patients with PD.

Previous clinical studies have shown electrocardiographic PWD to be a predictor of paroxysmal atrial fibrillation [10]. PWD reflects inhomogeneous atrial conduction via variation in p-wave duration between different surface ECG leads [18, 19]. PWD was significantly prolonged in PD patients in our study. Furthermore, we demonstrated that PWD had significant correlations with left intra-atrial and interatrial EMD durations in PD patients.

Patients with atrial fibrillation have an increased risk of stroke even after adjustment for risk factors [5]. PD has also been related with an increased risk of stroke [1–3]. Therefore, prediction of development of atrial fibrillation might be vital for risk stratification of PD patients with regard to ischemic stroke. Indeed, prolonged atrial conduction times were found to be significantly correlated with the severity of PD in our study.

Several potential explanations may be postulated for the pathophysiologic mechanisms by which atrial fibrillation and PD may be related. First, both atrial fibrillation and PD are associated with an increased inflammatory state [20–23]. Neuroinflammation plays an important role in the pathogenesis of PD and contributes to the progressive loss of nigral dopaminergic neurons [20]. Inflammatory responses mediated by activated glial cells, T cell infiltration, and increased expression of inflammatory cytokines are described as features of PD [20]. Likewise, the prevalence and prognosis of atrial fibrillation have been associated with increased levels of inflammatory markers [21, 22]. Moreover, high-sensitivity C-reactive protein levels have shown significant positive correlations with stroke risk factors in atrial fibrillation patients and have been associated with a composite endpoint of ischemic stroke, myocardial infarction, and death [23]. Second, oxidative stress plays an important role in the pathogenesis of atrial fibrillation and PD [24, 25]. Oxidative stress has been shown to increase dopamine cell degeneration in PD [24]. Increased oxidative stress measured by the redox potentials of glutathione has been found to be an independent predictor of atrial fibrillation after adjustment for risk factors, heart failure, coronary artery disease, and high-sensitivity C-reactive protein levels [25]. Third, dysfunction of the autonomic nervous system is a common occurrence in atrial fibrillation and PD [26–29]. Symptoms of cardiovascular dysautonomia such as orthostatic hypotension have been frequently reported in PD [26]. Likewise, increased vagal tone has been related to the onset of paroxysmal atrial fibrillation both through cholinergic and noncholinergic pathways [27, 28]. An interaction between sympathetic and parasympathetic nervous system demonstrated via recording activities from stellate ganglia and vagal nerve has been shown to play a role in the development of atrial fibrillation [29].

A limitation of our study might be that the study population was not followed up in terms of development of atrial fibrillation. Another limitation might be that data regarding inflammatory and oxidative markers were not studied. Also because of the limited number of patients included in the study, our findings require validation and further studies with larger patient groups with follow-up for arrhythmias are needed.

In conclusion, the present study showed that atrial conduction times were prolonged in patients with newly diagnosed PD. Furthermore, atrial conduction parameters were significantly correlated with the severity of PD. Our findings might contribute to risk stratification of PD patients with regard to atrial fibrillation.

## Figures and Tables

**Table 1:** Baseline demographic and clinical data of the study patients.

Characteristics	PD (*n*=51)	Control (*n*=31)	*p* value
Age, years	68.1 ± 10.4	67.2 ± 13.5	0.819
Male, *n* (%)	36 (71)	16 (52)	0.135
Hyperlipidemia, *n* (%)	12 (24)	8 (26)	0.816
DM, *n* (%)	8 (16)	5 (16)	0.958
Hypertension, *n* (%)	14 (27)	11 (35)	0.604
Smoking, *n* (%)	9 (18)	4 (13)	0.796
BMI (kg/m^2^)	23.5 ± 2.7	23.0 ± 2.5	0.356
BSA (m^2^)	1.94 ± 0.1	1.93 ± 0.12	0.659

*Medications*			
ASA, *n* (%)	6 (12)	5 (16)	0.820
ACEI, *n* (%)	5 (10)	5 (16)	0.617
ARB, *n* (%)	5 (10)	7 (23)	0.206
Calcium channel blocker, *n* (%)	7 (14)	5 (16)	0.765
Diuretic, *n* (%)	7 (14)	8 (25)	0.281
Statin, *n* (%)	8 (16)	6 (19)	0.900
OAD, *n* (%)	5 (10)	5 (16)	0.617
Insulin, *n* (%)	4 (8)	3 (10)	0.773
SBP (mmHg)	132.7 ± 19.8	135.5 ± 14.1	0.498
DBP (mmHg)	78.9 ± 8.4	83.1 ± 12.1	0.065
HR (bpm)	80 ± 11	79 ± 14	0.750
PWD (ms)	44.9 ± 6.1	40.0 ± 5.5	<0.001

PD, Parkinson's disease; DM, diabetes mellitus; BMI, body mass index; BSA, body surface area; ASA, acetylsalicylic acid; ACEI, angiotensin converting enzyme inhibitor; ARB, angiotensin receptor blocker; OAD, oral antidiabetic; SBP, systolic blood pressure; DBP, diastolic blood pressure; HR, heart rate; PWD, p-wave dispersion.

**Table 2:** Echocardiographic data and atrial conduction time intervals of the study patients.

Characteristics	PD (*n*=51)	Control (*n*=31)	*p* value
LVDD (mm)	44.6 ± 4.2	45.6 ± 2.9	0.232
LVSD (mm)	26.5 ± 4.9	27.2 ± 3.3	0.481
IVS (mm)	9.9 ± 1.9	9.3 ± 1.6	0.137
PW (mm)	9.1 ± 1.3	8.8 ± 0.8	0.179
LV EF (%)	60 (6)	60 (5)	0.782
LA volume index (cm^3^/m^2^)	17.1 ± 6.4	13.6 ± 2.5	0.006
DT (ms)	277 (91)	239 (67)	0.058
IRT (ms)	98.5 ± 15.9	102.6 ± 17.7	0.279
E/A ratio	0.9 ± 0.4	1.1 ± 0.4	0.057

*Atrial conduction times*			
ML-PA (ms)	63.1 ± 15.0	49.5 ± 9.0	<0.001
MS-PA (ms)	47.3 ± 9.9	35.0 ± 7.3	<0.001
TL-PA (ms)	55 (25)	37 (10)	<0.001
Intra-LA EMD (ms)	19 (10)	14 (8)	<0.001
Intra-RA EMD (ms)	15 (15)	5 (7)	<0.001
Interatrial EMD (ms)	18 (19)	13 (12)	0.002

LVDD, left ventricular diastolic diameter; LVSD, left ventricular systolic diameter; IVS, interventricular septum thickness; PW, posterior wall thickness; LV, left ventricle; EF, ejection fraction; LA, left atrium; A, mitral inflow late diastolic velocity; E, mitral inflow early diastolic velocity; DT, left ventricular deceleration time; IRT, isovolumic relaxation time; ML-PA, mitral lateral annulus PA duration; MS-PA; mitral septal annulus PA duration; TL-PA, tricuspid lateral annulus PA duration; EMD, electromechanical delay; RA, right atrium.

**Table 3:** Multivariate analyses of variables associated with Parkinson's disease.

Variables	OR	95% CI	*p* value
PWD	1.13	1.02–1.25	0.017
LA volume index	1.19	1.02–1.37	0.021
Intra-LA EMD	1.12	1.01–1.24	0.041
Interatrial EMD	1.08	1.01–1.16	0.026
Intra-RA EMD	1.15	1.05–1.25	0.001

OR, odds ratio; CI, confidence interval; PWD, p-wave dispersion; LA, left atrium; EMD, electromechanical delay; RA, right atrium.

## Data Availability

The data used to support the findings of this study are available from the corresponding author upon request.
